# Effects of drying methods and solvent extraction on quantification of major bioactive compounds in pomegranate peel waste using HPLC

**DOI:** 10.1038/s41598-022-11881-7

**Published:** 2022-05-14

**Authors:** Nishant Kumar, Pratibha, Neeraj, Rokayya Sami, Ebtihal Khojah, Amani H. Aljahani, Amina A. M. Al-Mushhin

**Affiliations:** 1 National Institute of Food Technology Entrepreneurship and Management (NIFTEM), Plot No. 97, Sector- 56, HSIIDC, Industrial Estate, Kundli, Sonipat, Haryana 131028 India; 2 National Institute of Technology Delhi, Plot No. FA7, Zone, P1, GT Karnal Road, Delhi, 110036 India; 3 Department of Food Science and Nutrition, College of Sciences, Taif University, P.O. 11099, Taif, 21944 Saudi Arabia; 4 Department of Physical Sport Science, College of Education, Princess Nourah bint Abdulrahman University, P.O. Box 84428, Riyadh, 11671 Saudi Arabia; 5 Department of Biology, College of Science and Humanities in Al-Kharj, Prince Sattam Bin Abdulaziz University, Al-Kharj, 11942 Saudi Arabia

**Keywords:** Biochemistry, Biological techniques, Biotechnology

## Abstract

Bioactive compound characterization is an essential step for utilizing pomegranate peel waste as food and nutraceuticals ingredients. In the present investigation, the effects of different drying methods (freeze, tray-oven, and sun) and extraction solvents such as methanol, ethanol, water, acetone, and hexane were investigated on the extraction and recovery of major bioactive compounds (ellagic acid, gallic acid, quercetin, and punicalagin) of pomegranate peel for two pomegranate varieties (i.e., Bhagwa and Ganesh) using high-performance liquid chromatography (HPLC). The results indicated that the freeze dried pomegranate peel powder of both pomegranate varities potential to extraction higher amount of bioactive compounds with methanol as extraction solvent as compared to other drying methods and solvents. Freeze-dried peel powder of Bhagwa pomegranate showed a higher amount of gallic acid (32.2 mg/g), ellagic acid (13.6 mg/g), punicalagin (15.2 mg/g), and quercetin (2.5 mg/g) with methanol solvent as compared to the other extract of Bhagwa and Ganesh varieties. The basis on the results of the current study, it can be concluded that the freeze-drying method of drying pomegranate peel powder and methanol as an extraction solvent are effective to recover higher amounts of bioactive compounds that can be utilized in food and pharmaceutical sectors at commercial scale.

## Introduction

Pomegranate fruit is known as Superfruits due to its delicious taste. It contains approximately 48–50% waste of whole fruits after juice extraction, corresponding to the pomegranate peel waste^1,2^. The pomegranate peel is an important source for natural bioactive compounds such as ellagitannins, tannins, gallic acid, punicalagin, catechin, rutinpunicalin, kaempferol, luteolin, glycosides, and epicatechin among other phenolic compounds^3–7^. The gallic acid, ellagic acid, punicalagin, and quercetin are considered major bioactive compounds of pomegranate peel. These bioactive compounds are responsible for different biological activities such as antimicrobial, antioxidant, anticancer, antimutagenic, and anti-inflammatory and help reduce the risk of chronic and cardiovascular diseases^8–10^. Several researchers have reported the biological activities and functions of pomegranate peel^11,12^. However, the drying methods are significant factors for drying products to remove water and reduce the chemical reaction or enzymatic activities^13–17^. The drying methods can affect the quality attributes such as color, nutritional and phytochemical activities of products^18–20^. Various drying methods such as sun-drying, vacuum-drying, freeze-drying, oven-drying, air-drying etc. are used to dry the products^21^. Furthermore, freeze-drying is a potential method for extraction and higher recovery of bioactive compounds and other phytochemical from natural plant sources compared to other drying methods; however, the freeze-drying method is expensive compared to others but retains the higher quality of the products^22–25^.

In addition, the extraction of bioactive compounds from plant sources is considered the primary step, and the solvents are essential factors for the extraction and recovery of bioactive compounds. Several types of solvents, i.e., polar and non-polar, extract the bioactive and phenolic compounds from the plants. Generally, non-polar and low polar solvents are used to extract lipophilic compounds and pigments from the plant. However, the recovery of phenolic compounds, yield and their free radical scavenging activity, antimicrobial and other biological activities of pomegranate peels and other plants depend on the types of solvents and extraction procedure^26–30^. Numerous studies Mphahlele et al.,^9^; John et al.,^31^; Ngo et al.,^32^; Buitrago et al.,^33^ have reported that drying and solvent have impacted the extraction and recovery of the bioactive compounds from pomegranate peel. Moreover, the HPLC study of major polyphenolic composition i.e. gallic acid, ellagic acid, punacalagin, and quercetin etc. of pomegranate peels has been extensively studied.

## Materials and methods

### Study period

The experiments were conducted from January 2018 to December 2018 at the National Institute of Food Technology Entrepreneurship and Management (India) and Sophisticated Industrial Materials Analytic (SIMA) Lab Pvt. Ltd. Delhi, India.

## Materials

The fresh pomegranate fruits were procured from the pomegranate orchard, Kullu, Himachal, through the National Research Center on Pomegranate (NRCP-ICAR), Solapur, Maharashtra (India) during the period of Dec. 2017–Jan. 2018. The study complies with local and national guidelines.

### Chemical and reagents

The analytical grades of chemicals, reagents, and standards were purchased from Sigma Aldrich Inc. and Hi-Media, India.

### Preparation of peel powder

**Figure 1:**
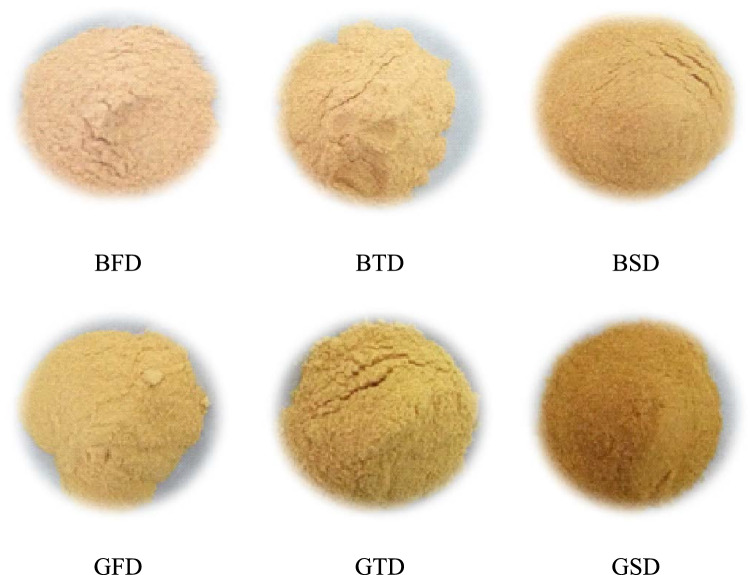
Different dried pomegranate peel powders. where *BFD* Bhagwa freeze-dried, *BTD* Bhagwa tray-dried, *BSD* Bhagwa sun-dried, *GFD* Ganesh freeze-dried, *GTD* Ganesh tray-dried, *GSD* Ganesh sun-dried.

The pomegranates were peeled manually to obtain the peel. The peel obtained was subjected to different drying methods to obtain peel powders (PGP). The blanching of obtained fresh pomegranate peel was carried out in a water bath at 90 °C for 30 s to remove surface impurities and contamination and dried under three different conditions viz., freeze-drying (− 45 °C for 32 h), tray-oven drying (60 °C for 29 h) and sun-dried (72 h) respectively (Fig. 1).

### Ultrasonic extraction of pomegranate peel

The dried peel powder obtained from the peels of Bhagwa and Ganesh was used for the extraction of phenolic and flavonoid content using polar (viz., methanol, ethanol, water, acetone) and non-polar solvents (hexane) through ultra-sonic assisted extraction method. The fine powder samples (0.2 g) of pomegranate peel of both varieties were sonicated in 10 ml of different solvents using an ultrasonic bath (CUB-5, Citizen, 40 kHz, 220-240 V, India) for 30 min at 45 °C temperature^10^. The prepared solution was centrifuged (Sigma, 3–18, KS, Germany) at 5 °C for 10 min with 8654 RPM and filtered using Whatman No. 11 filter paper to obtain the transparent extract. In total, there were three pomegranate peel powder samples for each variety prepared through three different drying methods (freeze-drying, tray-drying, and sun-drying). For each type of powder, five solvents (methanol, ethanol, water, acetone, and hexane) were used to prepare extracts. The peel extracts were later used for estimation of total phenolic content, total flavonoid content, antioxidant and antibacterial activity. The quantification of major phenolic content in the pomegranate peel of both varieties was also done by HPLC analysis. The 15 types extract of pomegranate peel powder for each variety was constructed with a completely randomized design (CRD) of research.

### HPLC analysis of samples

The identification and quantification of the major bioactive compounds i.e., gallic acid, ellagic acid, punicalagin, and quercetin etc. from pomegranate peel extract were determined using analytical reverse phase HPLC_4D UV (Agilent, 1200, HPLC Infinity) method followed by Elango et al.^34^ and Venkataramanamma et al.^35^ with minor modifications. HPLC column (C-18, Length- 250 × 4.6 mm id) with pore size of 5 μm was used for investigation at 260 nm UV wavelength by UV-PDA detector. The column speed for auxiliary draw and eject was set at 200 and 400μL/min, respectively with a constant flow rate (0.8). Solvent A (0.12 w/v) potassium dehydrate phosphate buffer with water at pH 1.2 and Solvent B acetonitrile was used to the investigation. The details about the gradient program for solvents (A & B) of HPLC are summed up in Table 1.

**Table 1:** Gradient programmode for isolation of the bioactive compounds of pomegranate peel extract.

Time (min)	Solvent (A) %	Solvent (B) %
0	95	5
15	45	55
20	30	70
25	95	5

### Identification and quantification of compounds

HPLC chromatogram was used to identification and quantification of bioactive compounds in pomegranate peel extract by retention time (Rt), area and height respectively. The results of bioactive compounds of each sample are expressed as mg/g of extract.

### Statistical analysis

Completely randomized design (CRD) was used to perform experimental work. All the experiments were analyzing in a replicate of three and average value with standard deviation (SD) was expressed as results. The drying methods and types of solvents were using as independent variables for the current study. Data were statistically analyzed using ANOVA and Post Hoc triplicate range test at P < 0.05 significance level by IBM SPSS software (24.0). Origin Pro (2019b) was used to graphically representation of average data.

## Results

### HPLC chromatogram of standards

The chromatograms of phenolic compounds standards such as ellagic acid, gallic acid, quercetin, and punicalagin in terms of retention time, area, and height are shown in Table 2. The maximum area was gallic acid with a 7.25 min retention time, followed by ellagic acid with 42.05% area and 53.38% height with 4.077 retention time. In the standard chromatogram, punicalagin showed minimum area (0.32%), height (0.47%) with a 14.77 min retention time. A punicalagin standard had the maximum retention time with the lowest area and height. The ellagic acid standard recorded the maximum height as compared to others. The results of chromatograms of the standards are supported by the previous studies done by Qu et al.^36^; Farag et al.^37^; Singh et al.^38^.

**Table 2:** HPLC chromatograms of phenolic standards (ellagic acid, gallic acid, quercetin, and punicalagin).

Compounds	Retention time (min.)	Area (%)	Height (%)
Ellagic acid (C_14_H_6_O_8_)	4.07	42.05	53.38
Gallic acid (C_7_H_6_O_5_)	7.25	50.57	38.69
Quercetin (C_15_H_10_O_7_)	14.36	7.05	7.45
Punicalagin (C_34_H_22_O_2_)	14.77	0.32	0.47

### Ellagic acid

The results pertaining to the extraction of ellagic acid from three different types of pomegranate peel powders extract have been presented in Table3. The ellagic acid compound was estimated through HPLC and has been expressed as mg/g of peel powder. The results indicated a significant impact of specific solvent used to extract the ellagic acid in both Bhagwa and Ganesh peel powders. Apart from solvents used, there was also a significant impact of drying to prepare the peel powders on ellagic acid content. Among various solvents used, extracts prepared with methanol solvent showed the highest ellagic acid content in all the three types of peel powders (freeze-dried, tray-dried, and sun-dried) followed by ethanol, water (control), and acetone in both the pomegranate varieties. The least ellagic acid content was recovered with hexane being used as an extraction solvent. The ellagic acid content recovered using different solvents were also statistically significant with respect to each other for a specific type of drying treatment. In the peel powder of Bhagwa, the statistically significant and maximum ellagic acid content (32.20 ± 0.01 mg/g) was recorded in freeze-dried peel powder. This ellagic acid content was also statistically significant and highest compared to all other solvents and drying treatments.

**Table 3:** Quantification of ellagic acid (mg/g) from pomegranate peel.

Bhagwa			
Solvents	BFD	BTD	BSD
M	32.20 ± 0.01^a^_l_	28.40 ± 0.02^b^_l_	7.50 ± 0.01^c^_l_
E	30.19 ± 0.01^a^_m_	19.60 ± 0.02^b^_m_	7.40 ± 0.01^c^_m_
W (c)	17.30 ± 0.01^a^_n_	14.30 ± 0.01^b^_n_	2.30 ± 0.01^c^_n_
A	14.20 ± 0.10^a^_o_	10.80 ± 0.00^b^_o_	1.40 ± 0.02^c^_o_
H	8.40 ± 0.02^a^_p_	4.80 ± 0.01^b^^p^	0.59 ± 0.02^c^_p_

Ganesh			
Solvents	GFD	GTD	GSD
M	17.50 ± 0.02^a^_l_	6.00 ± 0.10^b^_l_	5.30 ± 0.02^c^_l_
E	14.98 ± 0.2^a^_m_	5.10 ± 0.10^b^_m_	4.60 ± 0.02^c^_m_
W	3.30 ± 0.02^a^_n_	2.90 ± 0.02^b^_n_	1.80 ± 0.03^c^_n_
A	2.90 ± 0.00^a^_o_	1.30 ± 0.03^b^_o_	1.10 ± 0.00^c^_o_
H	1.80 ± 0.10^a^_p_	1.30 ± 0.01^b^_o_	0.10 ± 0.00^c^_p_

Mean ± SD n = 3, a, b, c represents a statistically significant difference between drying methods in row wise manner for specific solvent whereas l, m, n, o, p, q refers to statistically significant difference among solvents for respective drying method (column wise); *BFD* Bhagwa freeze-dried, *BTD* Bhagwa tray-dried, *BSD* Bhagwa sun-dried, *GFD* Ganesh freeze-dried, *GTD* Ganesh tray-dried, *GSD* Ganesh sun-dried, *M* methanol, *E* ethanol, *W* water, *A* acetone, *H* hexane), water was used as control.

The second-best result was obtained with ethanol solvent in freeze-dried powder (30.19 ± 0.01 mg/g) followed by methanol solvent in tray-dried powder (28.40 ± 0.02 mg/g). Among various drying treatments, the statistically significant and highest ellagic acid content was recovered in freeze-dried powder, followed by tray-dried and sun-dried peel powders. The least ellagic acid content was retrieved in sun-dried peel powder. The ellagic acid content observed in different drying treatments was also statistically significant with respect to each other for specific types of solvent. In the peel powder of Ganesh, the statistically significant and maximum ellagic acid content (17.50 ± 0.02 mg/g) was recorded in freeze-dried peel powder. This ellagic acid content was also statistically significant and highest compared to all other solvents and drying treatments. The second-best result was obtained with ethanol solvent in freeze-dried powder (14.98 ± 0.2 mg/g). It was also observed that the ellagic acid content was statistically at par for tray-dried peel powders with hexane and acetone as solvents (1.30 ± 0.03 mg/g, 1.30 ± 0.01 mg/g, respectively). It was also noted that in peel powder of Ganesh variety, irrespective of the drying treatments, the ellagic acid content was comparatively very low in acetone, hexane, and control (water) solvents as compared to methanol and ethanol.

The obtained results showed that methanol and ethanol could be used for the extraction of ellagic acid compounds from peel powder. The freeze-drying method and methanol solvent are significantly accounted to recover a higher amount of ellagic acid from pomegranate peel than respective drying treatments and solvents. The hexane solvent was not detected to quantify biological compounds from pomegranate peel powder due to lower efficiency to extract high polar compounds^17,39^.

### Gallic acid

The phenolic compound such as gallic acid passes a broadspectrum of biological activities, including phenolic, antioxidant, and antibacterial activities, etc.^40^ The results pertaining to the extraction of gallic acid content from three different types of pomegranate peel powders obtained from Bhagwa and Ganesh varieties have been presented in Table4. The results indicated a significant impact of the specific solvent used to extract the gallic acid bioactive compound in both Bhagwa and Ganesh peel powders. Apart from solvents used, there was also a significant impact on the method of drying to prepare the peel powders on gallic acid. Among various solvents used, extracts prepared with methanol solvent showed the highest amount of gallic acid in all the three types of peel powders, followed by ethanol, water, and acetone in both the pomegranate varieties. The least gallic acid content was recovered with hexane as an extraction solvent. The gallic acid bioactive compound recovered using different solvents were also statistically significant concerning each other for a specific type of drying treatment.

**Table 4:** Quantification of Gallic acid(mg/g) compounds from pomegranate peel extract.

Bhagwa			
Solvents	BFD	BTD	BSD
M	16.40 ± 0.02^a^_l_	11.60 ± 0.03^b^_l_	2.50 ± 0.02^c^_l_
E	16.20 ± 0.10^a^_m_	10.30 ± 0.01^b^_m_	1.60 ± 0.01^c^_m_
W	13.60 ± 0.02^a^_n_	9.90 ± 0.01^b^_n_	0.90 ± 0.10^c^_n_
A	8.45 ± 0.03^a^_o_	2.40 ± 0.01^b^_o_	0.03 ± 0.00^c^_o_
H	5.40 ± 0.01^a^_p_	0.80 ± 0.02^b^_p_	ND

Ganesh			
Solvents	GFD	GTD	GSD
M	10.90 ± 0.03^a^_l_	2.00 ± 0.10^b^_l_	0.80 ± 0.04^c^_l_
E	1.90 ± 0.03^a^_m_	1.10 ± 0.01^b^_m_	0.70 ± 0.02^c^_m_
W	1.30 ± 0.02^a^_n_	0.60 ± 0.02^b^_n_	0.30 ± 0.00^c^_n_
A	1.20 ± 0.02^a^_o_	0.40 ± 0.02^b^_o_	ND
H	0.80 ± 0.02^a^_p_	ND	ND

Mean ± SD n = 3, a, b, c represents a statistically significant difference between drying methods in row wise manner for specific solvent whereas l, m, n, o, p, q refers to statistically significant difference among solvents for respective drying method (column wise); *BFD* Bhagwa freeze dried, *BTD* Bhagwa tray-dried, *BSD* Bhagwa sun-dried, *GFD* Ganesh freeze-dried, *GTD* Ganesh tray-dried, *GSD* Ganesh sun-dried (*M* methanol, *E* ethanol, *W* water, *A* acetone, *H* hexane).

In the peel powder of Bhagwa, the statistically significant and maximum gallic acid content (16.40 ± 0.02 mg/g) was recorded in freeze-dried peel powder. This gallic acid content was also statistically significant and highest compared to all other solvents and drying treatments. The second-best result was obtained with ethanol solvent (16.20 ± 0.10 mg/g) followed by water as an extraction solvent in freeze-dried powder (13.60 ± 0.02 mg/g). The statistically significant and highest gallic acid content was recovered in freeze-dried powder followed by tray and sun dried peel powders among various drying treatments. The least gallic acid content was recovered in sun-dried peel powder. The gallic acid content as observed in different drying treatments was also statistically significant with respect to each other for a specific type of solvent. The least recovery of gallic acid from peel powder of Bhagwa pomegranate was found with hexane solvent (5.40 ± 0.01 mg/g) in freeze-dried followed by tray-dried (0.80 ± 0.02 mg/g) respectively. The peel powder obtained by sun-drying treatment was not found effective for recovering gallic acid compounds with hexane solvent. In the peel powder of Ganesh, the statistically significant and maximum gallic acid content (10.90 ± 0.03 mg/g) was recorded in freeze-dried peel powder. This amount of gallic acid content was also statistically significant and highest compared to all other solvents and drying treatments. The second-best result was obtained with methanol solvent in tray dried powder (2.00 ± 0.10 mg/g). It was also noted that in peel powder of Ganesh variety, irrespective of the drying treatments, the gallic acid content was comparatively very low in ethanol, water, acetone, and hexane solvents as compared to methanol. The peel powder extraction with hexane solvent showed gallic acid compound in freeze-dried (0.80 ± 0.02 mg/g). In peel powder obtained from tray and sun drying treatments with hexane solvent not detected gallic acid compounds. In peel powder obtained from tray-drying treatment also does not recovergallic acid with acetone as solvent. The results pertained that the maximum gallic acid was obtained with methanol as a solvent followed by ethanol in both the pomegranate varieties. The results also indicated that peel powders of Bhagwa variety recovered a significantly higher amount of gallic acid as compared to Ganesh. The results demonstrated that the freeze-drying method is significant potential to recover higher amounts of gallic acid as compared to other drying methods ^33^.

### Quercetin

The results of the quantification of quercetin from pomegranate peel powders are shown is presented in Table5. The results indicate a significant impact of the specific solvent used to extract the quercetin in both Bhagwa and Ganesh peel powders. Apart from solvents used, there was also a significant impact of drying to prepare the peel powders on quercetin content. Methanol as a solvent showed the highest quercetin, followed by ethanol, water, and acetone solvents in the Bhagwa and Ganesh varieties. The peel powder obtained from freeze-drying showed the statistically significant and highest amount of quercetin followed by tray and sun-dried peel powder obtained from both the Bhagwa and Ganesh varieties. In the peel powder of Bhagwa, the statistically significant and maximum quercetin (2.50 ± 0.01 mg/g) was estimated in freeze-dried peel powder. This quercetin was also statistically significant and highest compared to all other solvents and drying treatments.

**Table 5:** Quantification of Quercetin (mg/g) compounds from pomegranate peel extract.

Bhagwa			
Solvents	BFD	BTD	BSD
M	2.50 ± 0.01^a^_l_	0.55 ± 0.01^b^_l_	0.46 ± 0.00^c^_l_
E	1.40 ± 0.01^a^_m_	0.35 ± 0.02^b^_m_	0.12 ± 0.02^c^_m_
W	1.10 ± 0.10^a^_n_	0.07 ± 0.01^b^_n_	0.08 ± 0.00^b^_n_
A	0.06 ± 0.00^a^_o_	0.05 ± 0.00^a^_o_	ND
H	ND	ND	ND

Ganesh			
Solvents	GFD	GTD	GSD
M	0.53 ± 0.01^a^_l_	0.52 ± 0.58^a^_l_	0.10 ± 0.00^b^_l_
E	0.38 ± 0.00^a^_m_	0.18 ± 0.02^b^_m_	0.03 ± 0.01^c^_m_
W	0.31 ± 0.00^a^_n_	ND	ND
A	ND	ND	ND
H	ND	ND	ND

Mean ± SD n = 3, a, b, c represents a statistically significant difference between drying methods in row wise manner for specific solvent whereas l, m, n, o, p, q refers to statistically significant difference among solvents for respective drying method (column wise); *BFD* Bhagwa freeze-dried, *BTD* Bhagwa tray-dried, *BSD* Bhagwa sun-dried, *GFD* Ganesh freez-dried, *GTD* Ganesh tray-dried, *GSD* Ganesh sun-dried (*M* = methanol, *E* = ethanol, *W* = water, *A* = acetone, *H* = hexane).

The second-best result was obtained with ethanol solvent in freeze-dried powder (1.40 ± 0.01 mg/g) followed by water solvent in freeze-dried powder (1.10 ± 0.10 mg/g). Among various drying treatments, the statistically significant and highest quercetin content was recovered in freeze-dried powder followed by tray-dried and sun-dried peel powders. The least quercetin content was recovered in sun-dried peel powder. The quercetin content observed in different drying treatments was also statistically significant with respect to each other for a specific type of solvent. The peel powder extraction with hexane was not quantified the quercetin compounds in all drying treatment conditions. Quercetin compound was not detected with acetone solvent in peel powder obtained from sun-drying method.

In the peel powder of Ganesh, the statistically significant and maximum quercetin compound (0.53 ± 0.01 mg/g) was recorded in freeze-dried peel powder. This quercetin was also statistically significant and highest compared to all other solvents and drying treatments. The second-best result was obtained with methanol solvent in tray-dried peel powder (0.52 ± 0.58 mg/g). It was also noted that in peel powder of Ganesh variety, irrespective of the drying treatments, the quercetin compound was not detected in acetone and hexane solvents. The peel powder obtained from the tray and sun-drying treatments was also not show the quercetin compounds with water solvent. Overall results showed that the maximum quercetin was obtaining with methanol as solvent followed by ethanol in both the pomegranate varieties. The results also indicated that peel powders of Bhagwa variety had significantly higher recovery of quercetin than Ganesh. The methanol and ethanol solvent is accounted for suitable solvents for the quercetin compounds extraction compared to other solvents due to higher polarity and efficiency^41^.

### Punicalagin

The results pertaining to extraction and recovery of punicalagin compound from pomegranate peel powders are shown in Table 6. The punicalagin compound was estimated through HPLC and has been expressed as mg/g of peel powder. The results indicated a significant impact of the specific solvent used to extract the punicalagin in Bhagwa and Ganesh peel powders. Apart from solvents used, there was also a significant impact of drying to prepare the peel powders on punicalagin. Among various solvents used, extracts prepared with methanol solvent showed the highest punicalagin in all the three types of peel powders (freeze-dried, tray-dried, and sun-dried) followed by ethanol, water, and acetone in both the pomegranate varieties.

**Table 6:** Quantification of Punicalagin(mg/g) compounds from pomegranate peel extract.

Bhagwa			
Solvents	BFD	BTD	BSD
M	15.20 ± 0.20^a^_l_	11.20 ± 0.02^b^_l_	3.70 ± 0.01^c^_l_
E	13.80 ± 0.02^a^_m_	5.00 ± 0.20^b^_m_	1.40 ± 0.030^c^_m_
W	3.70 ± 0 .02^a^_n_	0.90 ± 0.02^b^_n_	0.80 ± 0.00^c^_n_
A	ND	ND	ND
H	ND	ND	ND

Ganesh			
Solvents	GFD	GTD	GSD
M	7.30 ± 0.01^a^_l_	5.13 ± 0.13^b^_l_	3.05 ± 0.02^c^_l_
E	4.60 ± 0.02^a^_m_	2.00 ± 0.05^b^_m_	0.46 ± 0.02^c^_m_
W	1.10 ± 0.01^a^_n_	0.10 ± 0.00^b^_n_	ND
A	ND	ND	ND
H	ND	ND	ND

Mean ± SD n = 3, a, b, c represents a statistically significant difference between drying methods in row wise manner for specific solvent whereas l, m, n, o, p, q refers to statistically significant difference among solvents for respective drying method (column wise); *BFD* Bhagwa freeze-dried, *BTD* Bhagwa tray-dried, *BSD* Bhagwa sun-dried, *GFD* Ganesh freeze-dried, *GTD* Ganesh tray-dried, *GSD* Ganesh sun-dried (*M* methanol, *E* ethanol, *W* water, *A* acetone, *H* hexane).

The least amount of punicalagin was recovered with acetone and hexane being used as an extraction solvent. The punicalagin compound recovered using different solvents were also statistically significant with respect to each other for a specific type of drying treatment. In the peel powder of Bhagwa, the statistically significant and maximum amount of punicalagin compound (15.20 ± 0.20 mg/g) was recorded in freeze-dried peel powder. This punicalagin compound was also statistically significant and highest compared to all other solvents and drying treatments. The second-best result was obtained with ethanol solvent in freeze-dried powder (13.80 ± 0.02 mg/g) followed by methanol solvent in tray-dried powder (11.20 ± 0.02 mg/g). Among various drying treatments, the statistically significant and highest punicalagin compound was recovered in freeze-dried powder followed by tray-dried and sun-dried peel powders. The most minor punicalagin compound was recovered in sun-dried peel powder. As observed in different drying treatments, the punicalagin compound was also statistically significant with respect to each other for a specific type of solvent. The peel powder obtained from all respective drying (freeze-drying, tray-drying, and sun-drying) methods were not recovered punicalagin compound with acetone and hexane solvent respectively. In the peel powder of Ganesh, the statistically significant and maximum recovery of punicalagin compound (7.30 ± 0.01 mg/g) was recorded in freeze-dried peel powder. This punicalagin content was also statistically significant and highest compared to all other solvents and drying treatments. The second-best result was obtained with ethanol solvent in freeze-dried powder (4.60 ± 0.02 mg/g). A similar trend of results was also observed in peel powder obtained from tray-drying method. It was also noted that the peel powder obtained from freeze-drying and tray-drying method was not recovered punicalagin compound with acetone and hexane solvent, respectively. In sun-dried peel powder extraction, the punicalagin compound was not recovered with water, acetone, and hexane solvent due to lower efficiency to extract phenolic compounds from pomegranate peel. The present study results are in line with a previous study done by Singh et al.^38^; those reported higher recovery of punicalagin compound with methanol as solvent followed by ethanol in both the pomegranate varieties. The results also indicated that peel powders of Bhagwa variety had a higher recovery amount of punicalagin compound than Ganesh. The results demonstrated that the freeze-drying method and methanol solvent for extraction had significant potential to retain and extract the higher amounts of punicalagin content compared to other drying methods and solvents.

In summary, the *Bhagwa *extract exhibited the most excellent quantity of phenolics such as gallic acid, punicalagin, quercetin, and ellagic acid compared with *Ganesh* extract. The methanolic extract exhibited the greatest amount of phenolics such as ellagic acid, gallic acid, and Punicalagin. The highest quantity of gallic acid was detected for *Bhagwa* in an aqueous solvent. The study reported that hexane and acetone solvents are not suitable for the phenolic extractions in pomegranate peels. Overall the freeze-drying method and methanol as an extraction solvent for extracting bioactive compounds from pomegranate peel are highly recommended. Further studies are needed to check the efficiency of a combination of solvents (polar/non-polar) for higher recovery of natural bioactive compounds from pomegranate peel waste and other natural sources for further application in food and pharmaceutical sectors at a commercial scale.

## Discussion

The freeze-drying method and methanol solvent are significantly accounted to recover a higher amount of ellagic acid from pomegranate peel than respective drying treatments and solvents. The hexane solvent was not detected to quantify biological compounds from pomegranate peel powder due to lower efficiency to extract high polar compounds^17,39^. The results have shown that the maximum gallic acid was obtained with methanol as a solvent followed by ethanol in both the pomegranate varieties. The results also indicated that peel powders of Bhagwa variety recovered a significantly higher amount of gallic acid than Ganesh. The results demonstrated that the freeze-drying method has significant potential for recovering the higher amounts of gallic acid compared to other drying methods^33^. Quercetin phenolic content was significantly higher obtained with methanol as solvent followed by ethanol in both the pomegranate varieties. The results also indicated that peel powders of Bhagwa variety had a higher recovery of quercetin significantly as compared to Ganesh. The methanol and ethanol solvent is accounted for suitable solvents for the extraction of quercetin compound as compared to other solvents due to higher polarity and efficiency^41^. In the case of punicalagin compounds, the sun-dried peel powder could not extract punicalagin compounds with water, acetone, and hexane solvent due to lower efficiency to extract phenolic compounds from pomegranate peel.

Overall results of the present investigation are supported by the previous findings reported by Mphahlele et al.^9^; John et al.^31^; Ngo et al.^32^; Buitrago et al.^33^; Qu et al.^36^; Farag et al.^37^; Singh et al.^38^ and Cheng et al.^17^. They reported the drying method directly impacted the recovery of bioactive compounds from pomegranate peel. The freeze-drying is the most desirable method to retain the higher amount of bioactive compounds such as ellagic acid, gallic acid, quercetin, and punicalagin of pomegranate peel. They also confirmed that the methanol as extract solvent has more potential to recover a higher amount of gallic acid, ellagic acid, quercetin and punicalagin content from pomegranate peel powder due to its high polar nature of methanol. The non-polar solvent such as hexane cannot recover the bioactive contents from pomegranate peel powders. Therefore, the study concluded that the freeze-drying methods and methanol as extractive solvent are potential to extract the phenolic compounds from pomegranate peel extract. The further study and practical implication of the pomegranate peel waste phenolic compounds should be explore in food and pharma sector.
